# Oral microbiome–metabolome axis links glycerophospholipid dysregulation to Alzheimer’s disease

**DOI:** 10.1080/20002297.2026.2732297

**Published:** 2026-09-29

**Authors:** Mengqi Jia, Runjuan Yang, Ying Xu, Zhenghua Wu, Jin Lu, Zuan Li, Qiying Yan, Guorong Fan, Yaxing Gui

**Affiliations:** a Department of Clinical Pharmacy, Shanghai General Hospital, Shanghai Jiao Tong University School of Medicine, Shanghai, P. R. China; b Department of Neurology, Shanghai General Hospital, Shanghai Jiao Tong University School of Medicine, Shanghai, P. R. China; c Department of Pharmacy, Shanghai Sixth People's Hospital, Shanghai Jiao Tong University School of Medicine, Shanghai, P. R. China

**Keywords:** Alzheimer’s disease, tongue biofilm, cerebrospinal fluid, UPLC-Q/TOF-MS, 16S rRNA sequencing

## Abstract

**Introduction:**

This study aimed to evaluate the feasibility of using tongue biofilm as a non-invasive, patient-friendly, and cost-effective biomarker source for identifying early microbial and metabolic disturbances associated with Alzheimer’s disease (AD).

**Materials and methods:**

A total of 62 outpatients were enrolled (31 with AD and 31 cognitively normal controls). Tongue biofilm samples were analyzed using 16S rRNA sequencing and untargeted metabolomics via UPLC-Q/TOF-MS. Additionally, cerebrospinal fluid (CSF) samples from 36 individuals (18 per group) were examined under identical untargeted metabolomics processing protocols to validate the metabolomic findings.

**Results and discussion:**

Microbiome analysis revealed an increased relative abundance of Proteobacteria in the AD group, while Firmicutes and Bacteroidetes were enriched in controls. Metabolomic profiling identified 88 significantly different metabolites between groups, 64 of which achieved area under the curve (AUC) values > 0.90 in the ROC analysis. Glycerophospholipid metabolism has emerged as a key dysregulated pathway in AD, a finding further corroborated by CSF metabolomic analysis. Tongue biofilm represents a non-invasive, cost-effective, and accessible biomarker source, offering potential for AD diagnosis and research when coupled with microbiome and metabolomic analyses. Moreover, the role of glycerophospholipid metabolism in AD pathogenesis warrants further investigation.

## Graphical abstract

**Figure:**
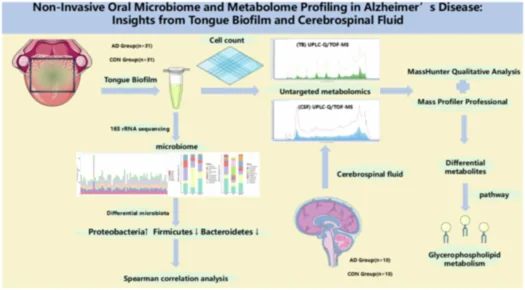

## Introduction

Alzheimer’s disease (AD) is a progressive and irreversible neurodegenerative disorder [1,2]. AD is the leading cause of dementia, affecting 50 million people worldwide in 2018, with numbers expected to triple by 2050, predominantly in low- and middle-income countries, making it one of the most costly and burdensome diseases of the 21st century [3]. AD progresses slowly, causing a significant loss of self-care ability and imposing psychological, physical, and economic burdens on both patients and their families. Unfortunately, it is often diagnosed only when clear symptoms appear, making early interventions less effective [4,5]. With the progression of global population ageing and the rising prevalence of AD, this will result in a costly disease burden [6]. Early screening and diagnosis are essential, but current clinical practices face major challenges in detecting early-stage AD [1]. The pathogenesis of AD is still unclear, with current theories focusing on beta-amyloid deposition, Tau protein abnormalities, and neuroinflammation. Diagnosis primarily relies on clinical symptoms and biomarkers, detected through MRI, PET imaging, Cerebrospinal Fluid (CSF), serum, and plasma [3]. The invasive risks of CSF lumbar puncture, along with the high cost of PET imaging, create significant clinical burdens. While FDA-approved blood biomarkers have advanced AD clinical screening, they primarily serve diagnostic endpoints and offer limited insight into the peripheral pathways driving central pathological progression. Therefore, there remains an urgent need for accessible biospecimens that can support large-scale cohort research while uncovering novel mechanistic links between peripheral dysregulation and AD pathogenesis.

During the clinical examination of patients, we observed that individuals with AD exhibited noticeable abnormalities in their tongue biofilm (TB), suCSFSFch as a thick white or yellowish, sticky coating. This observation sparked our interest. TB refers to the coating on the surface of the tongue and is an important diagnostic element in traditional Chinese medicine ‘inspection’ diagnosis. It is primarily composed of bacteria, keratinized and exfoliated epithelial cells, saliva, blood metabolites, and residual food particles [7–9]. Some scholars have confirmed the utility of tongue images [10–13], with certain studies validating their role as a stable diagnostic method for gastric cancer that is superior to traditional blood biomarkers. Research on TB is also in full swing, gradually expanding from oral diseases [14], gastrointestinal diseases [15–18] to a broader perspective [19,20]. There is literature indicating that TB has the potential to become a non-invasive diagnostic tool for disease detection and a tool for tracking prognosis [21,22]. TB sampling is easy, non-invasive, low-risk, and promising, making it suitable for community-based detection. With further research and improvement, it could allow patients to perform self-sampling for basic testing. We aim to study TB in AD patients to provide new sample materials and methods for AD research.

Current research on the microbiome-AD axis has increasingly focused on the intricate link between microbial communities and AD pathogenesis. Different from the widely reported gut‒brain axis, there are distinguished oral cavity–brain anatomical and physiological pathways that independently mediate the crosstalk between oral microorganisms and central nervous system lesions. Specifically, oral microbes and their metabolites can cross the blood–brain barrier and affect brain pathological changes through multiple pathways, including the trigeminal nerve conduction, systemic circulation translocation, and inflammatory signalling cascades. This unique oral–brain regulatory mechanism has been validated by multiple specialized studies focusing on the oral microbiome and AD [23–27]. Given that AD is widely recognized as a metabolic disorder, identifying metabolites associated with disease progression is essential not only for early detection and prevention but also for deepening our understanding of its underlying mechanisms and therapeutic strategies [28–30]. Notably, based on the close anatomical and physiological connection between the oral cavity and the brain, TB serves as an ideal biospecimen. Unlike periodontitis-associated subgingival plaque, which is highly susceptible to local inflammatory fluctuations, TB is a stable, high-biomass oral microecological niche. It accumulates more microbial metabolites than saliva and dental plaque, maintains consistent microbial-metabolic signatures independent of active periodontitis, and reflects both local microecological shifts and AD-related systemic inflammation. Furthermore, relative to CSF, TB is non-invasive and cost-effective. Therefore, we selected TB as our research target to explore novel microbial and metabolic biomarkers relevant to AD diagnosis within this emerging sample type. Consequently, in investigating the relationship between TB and AD, we specifically examined whether the microbial and metabolic profiles across different groups exhibited associative patterns consistent with previous findings. Non-targeted metabolomics analysis attempts to identify all peaks detected above the noise threshold and uses peak information for statistical analysis. This comprehensive analysis of metabolites detected in biological samples is mainly used for the discovery of clinically relevant biomarkers [31]. Ultra performance liquid chromatography–quadrupole-time of flight-mass spectrometry (UPLC-Q/TOF-MS) is characterized by its high resolution, high sensitivity, and good reproducibility. It is widely used in omics analyses, such as metabolomics and has played a significant role in various diseases and multiple sample types [32–36]. 16S Ribosomal RNA sequencing (16S rRNA sequencing) technology is widely used in microbiome research. It can elucidate the composition of microbial communities and is a reliable method for high-throughput sequencing analysis [37,38].

In summary, we plan to use the approaches of non-targeted metabolomics and microbiomics, employing UPLC-Q/TOF-MS and 16S rRNA sequencing methods, to analyze the TB of AD patients. We aimed to identify associations between the metabolic and microbial characteristics of TB and AD, filling a gap in this area of research. Simultaneously, we seek to identify metabolic differences between AD patients and healthy individuals, providing new pathways, methods, and ideas for exploring therapeutic biomarkers for AD. In addition, considering the status of CSF as the provisional gold standard in AD diagnostics, we included CSF metabolomics for a selected cohort of AD patients and healthy controls. By subjecting both sample types to the same experimental protocols, we sought to identify the concordance and divergence in their metabolic results, thereby further exploring the utility of TB as a promising sample for AD detection.

## Materials and methods

### TB sample collection

#### Source of samples

Participants comprising the AD group were recruited from the Neurology Outpatient Department of Shanghai General Hospital. The final sample size was contingent upon the number of eligible patients presenting within a single outpatient cycle. The control (CON) group was composed of the patients’ accompanying family members who satisfied the stipulated inclusion criteria. Ethical approval for this investigation was granted by the Ethics Committee of Shanghai General Hospital (Approval Nos. 2024HS158 and 2024HS037). Written informed consent was procured from all participants prior to their inclusion. Patients with intact decision capacity provided direct written informed consent. Patients with mild-to-moderate cognitive impairment who lacked full independent decision capacity were enrolled only after obtaining written surrogate consent from their primary legal caregiver, plus verbal assent from the patient themselves. All consent documents were double-signed by clinicians and researchers for archiving.

This study was conducted in accordance with the tenets of the Declaration of Helsinki and is registered in the Chinese Clinical Trial Registry (Identifier: ChiCTR2400091883).

#### Inclusion and exclusion criteria

Diagnostic criteria for AD: Diagnostic and Statistical Manual of Mental Disorders, Fifth Edition (DSM-5): (1) significant cognitive decline in ≥ 1 cognitive domain (memory, executive function, visuospatial, language); (2) cognitive impairment interferes with independent daily living; (3) no delirium; and (4) cognitive deficits cannot be fully explained by other psychiatric/neurological disorders. For clinical confirmation, we integrated neuropsychological scales (MMSE) and neuroimaging records as auxiliary diagnostic evidence.

The inclusion criteria for the AD group: (1) meet the diagnostic criteria for AD; (2) age > 60 years; and (3) voluntary participation with informed consent.

The inclusion criteria for the CON group: (1) confirmed absence of neurodegenerative diseases; (2) age > 60 years; (3) matched with the AD group in terms of the prevalence of hypertension/hyperlipidemia and corresponding medication use; and (4) voluntary participation with informed consent.

Exclusion criteria: (1) use of medications affecting TB status within two weeks prior to sampling (e.g. antibiotics, antifungal drugs, traditional Chinese medicine); (2) presence of conditions affecting TB status (e.g. acute upper respiratory tract infection, oral ulcers, glossitis and so on.); and (3) history of other systemic diseases (e.g. tumours, infectious diseases, or digestive disorders).

In addition to meeting clinical diagnostic criteria, the AD group had Aβ-42 levels < 650  pg/mL or an Aβ-42/Aβ-40 ratio ≤ 0.05.

#### Collection and processing of TB samples

To mitigate potential confounding effects from dietary residues and medications, participants were instructed to fast and abstain from AD-related pharmacological agents prior to sample collection. Upon arrival, each participant underwent a standardized oral cleansing protocol consisting of three successive mouth rinses with sterile physiological saline (0.9% NaCl) to remove exogenous debris and saliva. Subsequently, the TB was photographed in situ for documentation. TB specimens were then obtained by performing five consecutive scrapings across the dorsum of the tongue, from the left to the right lateral border. Each collected sample was immediately transferred to a sterile Eppendorf tube containing 500  μL of normal saline (0.9% NaCl, to preserve microbial cell membrane integrity during scraping and homogenization), thoroughly homogenized by pipetting, and subjected to cell enumeration using a Countstar Mira BF-S automated cell counter. The cellular concentration was then standardized to 10^6^ cells/mL, and all samples were archived at −80 °C pending subsequent metabolomic analysis. Each standardized tongue scraping homogenate was split into two independent 250  μL aliquots immediately after cell counting: Aliquot 1 was stored at −80 °C for DNA extraction and 16S rRNA microbiome sequencing; Aliquot 2 underwent metabolite extraction and UPLC-Q/TOF-MS analysis. No cross-contamination occurred between the two omics workflows.

For metabolite extraction, a 200 μL aliquot of each homogenized sample was centrifuged at 1,000  rpm for 4  min at 4 °C. The resulting supernatant was carefully decanted and evaporated to dryness under a gentle stream of nitrogen gas. The resulting cell pellet was re-suspended in 100  μL of ice-cold 90% acetonitrile and subjected to three successive freeze‒thaw cycles (−80 °C for 10  min per cycle) to ensure complete cellular lysis. The volume was then adjusted to 200 μL with the addition of 90% acetonitrile, followed by vigorous vortexing to achieve a homogeneous lysate. To further disrupt the cellular components and enhance metabolite recovery, the lysate was sonicated for 10 cycles (30 s per cycle). Finally, the mixture was clarified by centrifugation at 15,000 rpm for 10 min at 4 °C, and 150 μL of the clear supernatant was harvested for downstream analytical processing.

### Collection and processing of CSF samples

Samples were sourced from inpatients in the Department of Neurology at Shanghai General Hospital. After thawing 50 µL of the sample, 450 µL of acetonitrile was added to achieve a final concentration of 90% acetonitrile, matching the metabolite extraction conditions used for TB samples.

### UPLC-Q/TOF-MS processing procedure

Metabolite profiling was performed using an Agilent 1290 Infinity UPLC system (Milford, MA, USA) coupled to an Agilent 6545 Q-TOF MS/MS, equipped with a dual Agilent Jet Stream electrospray ionization (ESI) source operating in positive ion mode. Chromatographic separation was achieved on a Waters ACQUITY HSS T3 column (3.0 × 150 mm, 1.8 μm) maintained at 35 °C. The mobile phase, delivered at a flow rate of 0.3  mL/min, consisted of (A) 10 mM ammonium acetate with 0.1% formic acid and (B) acetonitrile. The gradient elution program was as follows: 10–50% B over 0–2 min; 50–95% B over 2–14.5 min; a return to 10% B from 14.5 to 15 min; and an equilibration at 10% B from 15 to 20 min. The injection volume for each analysis was 5  μL. For mass spectrometry detection, full-scan MS spectra were acquired over a range of 100–1,000 m/z, and data-dependent MS/MS spectra were collected from 50 to 800 m/z. The fragmentor voltage was set to 175 V, and the collision energies were alternated between 10 and 30 eV for fragmentation. The key source parameters were optimized as follows: gas temperature at 320 °C, sheath gas temperature at 350 °C, sheath gas flow at 11 L/min, nebulizer gas flow at 8 L/min, and a nebulizer pressure of 35 psig.

Prior to analysis, all the samples were thawed from −80 °C storage and equilibrated to room temperature. A quality control (QC) sample was prepared by pooling a 20 µL aliquot from each individual sample; this QC sample underwent identical preprocessing and was analyzed alongside the experimental samples. The analytical run sequence was initiated with the AD group, followed by the control (CON) group. To monitor and correct for instrumental drift and analytical variability, the QC sample was injected at intervals of every 10 samples. Furthermore, a pre-injection of 20% acetonitrile was performed before each sample batch to confirm baseline stability.

### Processing procedure for 16S rRNA sequencing

Total genomic DNA was extracted from TB samples using the MagBeads FastDNA Kit for Soil (116564384; MP Biomedicals, CA, USA) following the manufacturer’s standard protocols.

DNA extraction quality control: The concentration and purity of extracted genomic DNA were measured using a NanoDrop NC2000 spectrophotometer (Thermo Fisher Scientific, Waltham, MA, USA). The samples were considered qualified with an A260/A280 ratio ranging from 1.8 to 2.0 and a DNA concentration of no less than 20 ng/μL. DNA integrity was further verified via 1% agarose gel electrophoresis, and samples with obviously degraded high-molecular-weight DNA bands or severe nucleic acid contamination were excluded from subsequent experiments. The V3–V4 hypervariable region of the bacterial 16S rRNA gene was amplified by polymerase chain reaction (PCR) using sample-specific barcoded primers: forward primer 338F (5'-ACTCCTACGGGAGGCAGCA-3') and reverse primer 806R (5'-GGACTACHVGGGTWTCTAAT-3'). PCR amplicons were purified with VAHTS DNA Clean Beads (Vazyme, Nanjing, China) and preliminarily quantified using a microplate reader.

Library preparation quality control: Sequencing libraries were constructed using the TruSeq Nano DNA LT Library Prep Kit (Illumina, San Diego, CA, USA). The fragment size distribution of each library was detected using an Agilent 2100 Bioanalyzer (Agilent Technologies, Palo Alto, CA, USA), with a target insert size of approximately 460 bp. The absolute concentration of each library was accurately quantified with the Quant-iT PicoGreen dsDNA Assay Kit (Invitrogen, Carlsbad, CA, USA), and all qualified libraries were normalized to equal molar concentrations before pooling.

Sequencing platform and run quality control: Qualified pooled libraries were sequenced on the Illumina NovaSeq 6000 platform with a paired-end 250 bp (PE250) sequencing strategy using the NovaSeq 6000 SP Reagent Kit (500 cycles). A 10% volume of PhiX control library was spiked into the sequencing run to monitor the sequencing error rate and base calling accuracy. Raw reads with a Q20 base accuracy rate below 90% were filtered out during downstream bioinformatic processing to ensure data reliability.

### Data analysis

Quantitative data are expressed as the mean ± standard deviation (x̅ ± s), and count data are presented as numbers and percentages. Statistical analysis was performed using SPSS (version 25) and R (version 4.3.3). The experimental and data processing flow is shown in Figure 1.

**Figure 1.**
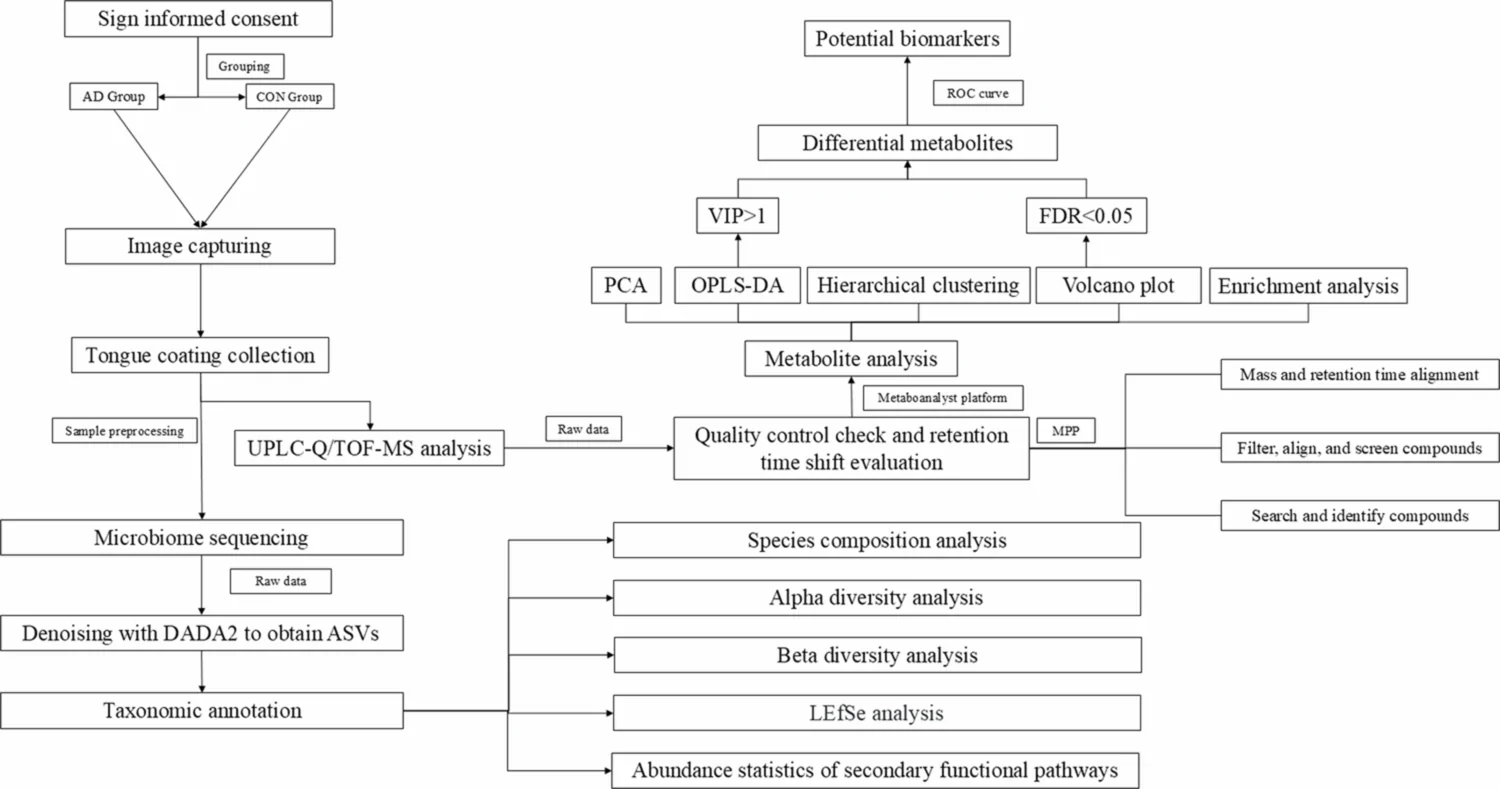
Workflow of the study.

UPLC-Q/TOF-MS data were processed using MassHunter Qualitative Analysis software (version 10.0) and Mass Profiler Professional (MPP) software (version 15.1). The workflow included quality control, retention time checks, feature detection, and gap-filling analysis. Compound identification was performed using METLIN and HMDB, with the processed data imported into MetaboAnalyst 6.0 for statistical analyses, including PCA, PLS-DA, and pathway enrichment. Differential metabolites with FDR < 0.05 and VIP > 1 values were further analyzed, with ROC analysis assessing diagnostic potential.

Raw 16S rRNA sequencing data were demultiplexed, and barcodes were removed, followed by denoising and Chimera removal using DADA2 in QIIME2 (version 2019.4). ASVs were generated, and singletons were excluded. Taxonomic annotation classified ASVs at the phylum and genus levels using the Greengenes 13.8 database (enables direct cross-literature comparison of phylum-level microbial shifts (Firmicutes/Bacteroidetes/Proteobacteria)), with alpha and beta diversity indices assessing microbial variation. LEfSe analysis identified significantly different taxa using non-parametric tests and LDA. For functional pathway prediction, ASV representative sequences were placed into the PICRUSt2 reference tree, and hidden-state prediction was performed to generate predicted enzyme commission (EC) numbers. EC annotations were subsequently mapped to the MetaCyc metabolic pathway database (v22.0) to infer pathway abundances. Differences in pathway abundance between the AD and CON groups were tested via the non-parametric Wilcoxon rank-sum test, with *P* < 0.01 defined as the significance threshold.

TB images were assessed using the tongue coating score (TCS) [39]. TCS is the product of area and thickness scores. The area score ranges from 0 (no coating) to 3 (>2/3 coverage). The thickness score ranges from 0 (no coating) to 3 (thick coating). This method quantifies TB severity by combining coverage and coating density.

TB microbiome–metabolome association analysis: Pairwise Spearman’s rank correlations were computed between differential microbes and metabolites, with FDR correction for multiple testing. The results were visualized as correlation heatmaps, where * denotes adjusted *P* < 0.05 and ** denotes adjusted *P* < 0.01.

TB–CSF metabolomic concordance analysis: Identical multivariate (PCA, OPLS-DA, permutation test) and pathway enrichment pipelines were applied to both the TB and CSF datasets. Shared and sample-specific dysregulated pathways were identified by comparing pathway impact scores and significance; concordant enrichment across biospecimens was interpreted as validation of core AD lipid pathological signals.

## Results

### Demographic characteristics

As shown in Table 1, in the TB group, the CON group had a mean age of 71.77 ± 6.453 years, with 54.8% females, while the AD group had a mean age of 74.29 ± 7.231 years, with 61.3% being females. There were no significant differences in age or sex distribution between the groups (age: t = −1.449, *p* = 0.153; sex: χ^2^ = 0.265, *p* = 0.607), suggesting no confounding effects. Regarding comorbidities, 48.4% of both groups had hypertension, and 48.4% had hyperlipidemia, with no significant differences (hypertension: χ^2^ = 0.065, *p* = 0.799; hyperlipidemia: χ^2^ = 0.000, *p* = 1.000). The median TCS was 4 (IQR: 2–6) in the AD group and 2 (IQR: 2–3) in the CON group, with a significant difference between groups (Z = 2.187, *p* = 0.029), indicating greater TB severity in AD patients. AFP and CEA are routine systemic tumour screening markers for our hospital’s neurology inpatient cohort. Per exclusion criteria, participants with malignant tumours were excluded; we reported these indicators to explicitly confirm that there were no intergroup differences in tumour risk markers, eliminating potential confounding from undiagnosed malignant disease that could alter systemic metabolism. No significant intergroup differences were observed for AFP and CEA (*P* > 0.05). All the subjects were recruited from a stable Shanghai cohort with similar backgrounds, consistently holding a secondary or vocational school education. Consequently, no significant difference in education years was observed between the groups. In addition, a demographic analysis of the CSF group is shown in Table 1.

### 16S rRNA sequencing results

As shown in Figures 1 and 2, 1,299 ASVs (8.89%) were shared between the CON and AD groups, with 7,185 unique to the CON group (49.19%) and 6,124 to the AD group (41.92%), indicating notable differences in microbial composition. Figure 2B highlights the relative abundance of dominant taxa, with Proteobacteria being most abundant in the AD group and Firmicutes and Bacteroidetes higher in the CON group. At the genus level, differences were observed in *Prevotella*, *Veillonella*, *Leptotrichia*, *Actinomyces*, *Streptococcus*, and *Neisseria*. Phylum-level comparisons (Figure 2C) revealed increased Proteobacteria and decreased Firmicutes in AD patients. Genus-level analysis (Figure 2D) showed higher *Neisseria* and lower *Streptococcus* and *Actinomyces* in AD (Supplementary data are available in Table S2). The alpha diversity indices (Figure 2E) did not differ significantly between the groups (*P* > 0.05). LEfSe analysis (Figure 2F) identified taxa enriched in the CON group, including *Veillonellaceae*, *Clostridiales*, and *Pseudomonas*, while the AD group was enriched in *Proteobacteria*, *Gammaproteobacteria*, and *Haemophilus*. Functional prediction (Figure 2G) indicated significant downregulation of PWY-6210 and PWY-5430 in the AD group (*P* < 0.01).

**Figure 2.**
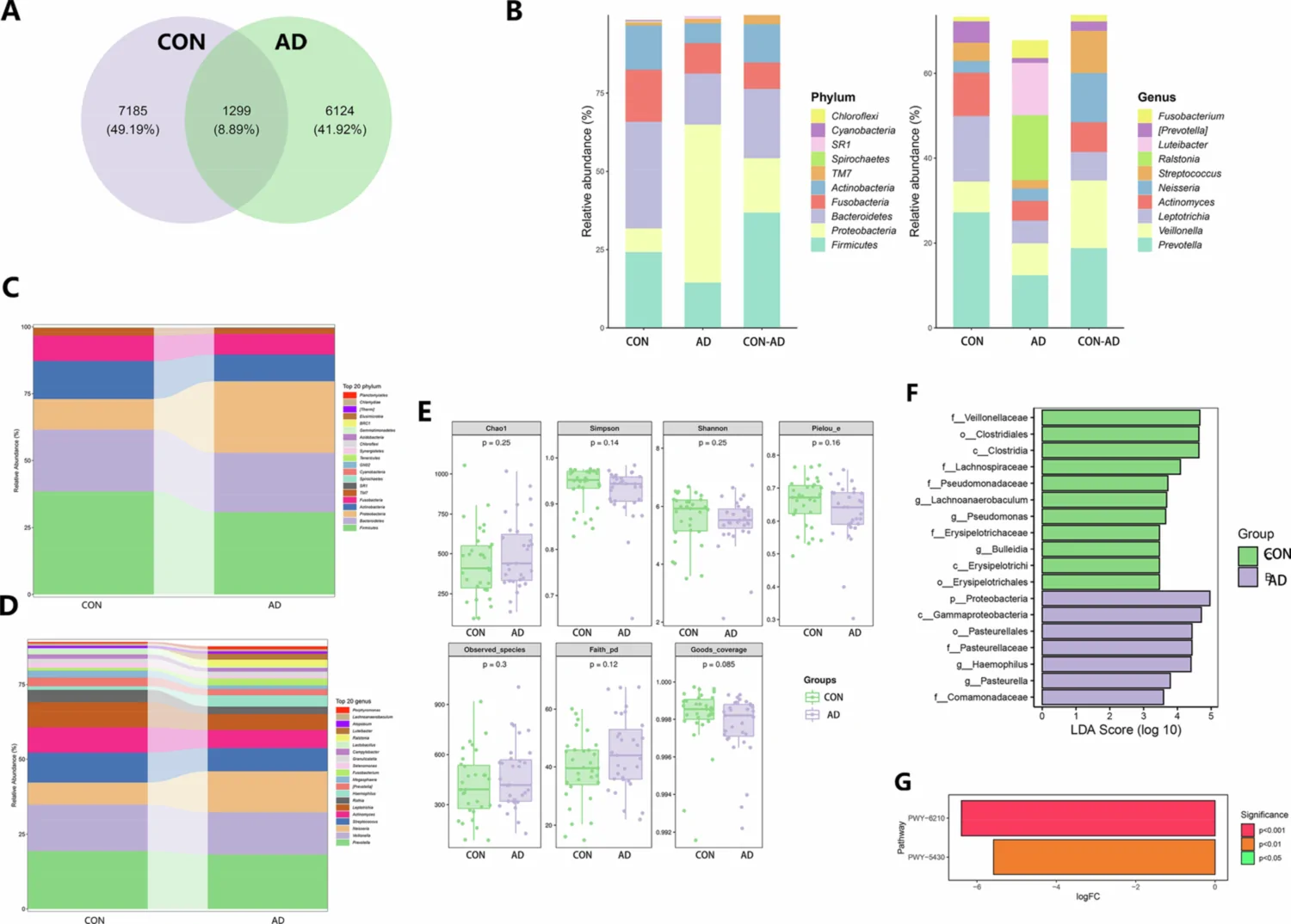
Microbial community structure and diversity analysis between the CON and AD groups. (A) Venn diagram showing unique and shared ASVs between the CON and AD groups. (B) Bar plots depicting the ASV taxonomic composition at the phylum and genus levels. (C) Relative abundance of microbial communities at the phylum level. (D) Relative abundance at the genus level, with the top 20 genera colour-coded and their proportions indicated by bar length. (E) Box plots comparing alpha diversity between groups, including Chao1, Simpson, Shannon, Pielou's evenness, observed species, and Faith’s phylogenetic diversity indices. (F) LEfSe analysis of differential taxa, with LDA scores indicating significance (LDA > 2). Green bars: CON-enriched taxa, purple bars: AD-enriched taxa. (G) Differentially abundant MetaCyc metabolic pathways between the groups.

**Table 1.** Demographic characteristics of the TC group.

Variable			CON	AD	Test statistic [RD (95%CI)]		*P*-value
TC group							
	Age (Mean ± SD)		71.77 ± 6.453	74.29 ± 7.231	*t* = −1.449	−2.516 (−5.991,0.958)	0.153
	Gender [*n* (%)]	Woman	17(54.8%)	19(61.3%)	χ^2^ = 0.265		0.607
		Man	14(45.2%)	12(38.7%)			
	TCS [M (P_25_, P_75_)]		2(2,3)	4(2,6)	*z* = 2.187	−2[−2, −1]	0.029
	BMI (Mean ± SD)		22.72 ± 2.367	22.04 ± 2.388	*t* = 1.123	0.678 [−0.530,1.886]	0.266
	Hypertension [*n* (%)]	Yes	16(51.6%)	15(48.4%)	χ^2^ = 0.065		0.799
		No	15(48.4%)	16(51.6%)			
	Hyperlipidemia [*n* (%)]	Yes	15(48.4%)	15(48.4%)	χ^2^ = 0.000		1.000
		No	16(51.6%)	16(51.6%)			
CSF group							
	Age (Mean ± SD)		66.50 ± 7.430	69.67 ± 5.584	*t* = −1.446	−3.167 [−7.631,1.298]	0.158
	Sex [*n* (%)]	Woman	8	13	OR = 3.25		0.176
		Man	10	5			
	BMI (Mean ± SD)		23.84 ± 3.03	22.54 ± 2.58	*t* = 1.380	1.295[−0.612,3.203]	0.177
	Aβ−42 (Mean ± SD)		1157.82 ± 271.97	492.80 ± 169.92	*t* = 8.798	665.019[510.310,819.729]	< 0.001
	Aβ−42/Aβ−40[M(P_25_,P_75_)]		0.083(0.078,0.089)	0.041(0.033,0.064)	*z* = 3.608	0.042 [0.023, 0.051]	< 0.001
	MMSE [M (P_25_, P_75_)]		27.5(27, 29)	23.5(16, 24)	*z* = −5.117	−4 [−9, −3]	< 0.001
	Total Cholesterol (Mean ± SD)		4.34 ± 1.08	4.50 ± 1.14	*t* = −0.411	−0.152[−0.902,0.598]	0.684
	Triglyceride (Mean ± SD)		1.44 ± 0.81	1.16 ± 0.42	*t* = 1.280	0.275[−0.162,0.712]	0.209
	HDL-C (Mean ± SD)		1.20 ± 0.29	1.48 ± 0.46	*t* = −2.172	−0.279[−0.541, −0.179]	0.037
	LDL-C [M (P_25_, P_75_)]		2.53(2.05,3.49)	2.46(2.00,3.31)	*z* = 0.253	0.040[−0.92,0.925]	0.800
	Fasting Glucose (Mean ± SD)		4.86 ± 0.89	5.23 ± 1.38	*t* = −0.965	−0.374[−1.161,0.413]	0.341
	HbA1c [M (P_25_, P_75_)]		5.7(5.53,6.28)	5.9(5.43,6.08)	*z* = 0.063	−0.1(−0.4,0.6)	0.949
	AFP [M (P_25_, P_75_)]		1.84(1.04,2.69)	1.34(0.67,1.76)	*z* = 1.582	0.508[−0.39,1.37]	0.114
	CEA (Mean ± SD)		2.85 ± 1.20	2.77 ± 1.66	*t* = 0.156	0.076[−0.908,1.059]	0.887

AFP = Alpha-fetoprotein; CEA = Carcinoembryonic antigen.

In the CSF group, except for Aβ-42 and Aβ-42/Aβ-40, the rest are the blood indicators of the corresponding personnel in the group.

### TB metabolomics data results

#### Data stability and reliability

As shown in Figure 3, both PCA (Figure 3A) and PLS-DA (Figure 3B) demonstrated clear separation among the AD, CON, and QC groups, indicating distinct group-specific patterns. The PLS-DA model showed high performance, with elevated accuracy, R^2^, and Q^2^ values, and a permutation test yielded a significant *p*-value of 0.0215 (Figure 3C and D), confirming the model's robustness. The QC samples clustered tightly, reflecting high data quality and stability. PCA and OPLS-DA further differentiated the metabolic profiles of the AD and CON groups (Figure 3E and F). A 2,000-time permutation test (Figure 3G) confirmed excellent model fitness (R^2^Y = 0.997) and predictive ability (Q^2^= 0.984), with *p*-values well below 0.05, indicating statistical significance and ruling out overfitting. The Q^2^ value exceeding 0.8 reinforced the model's strong predictive power.

**Figure 3.**
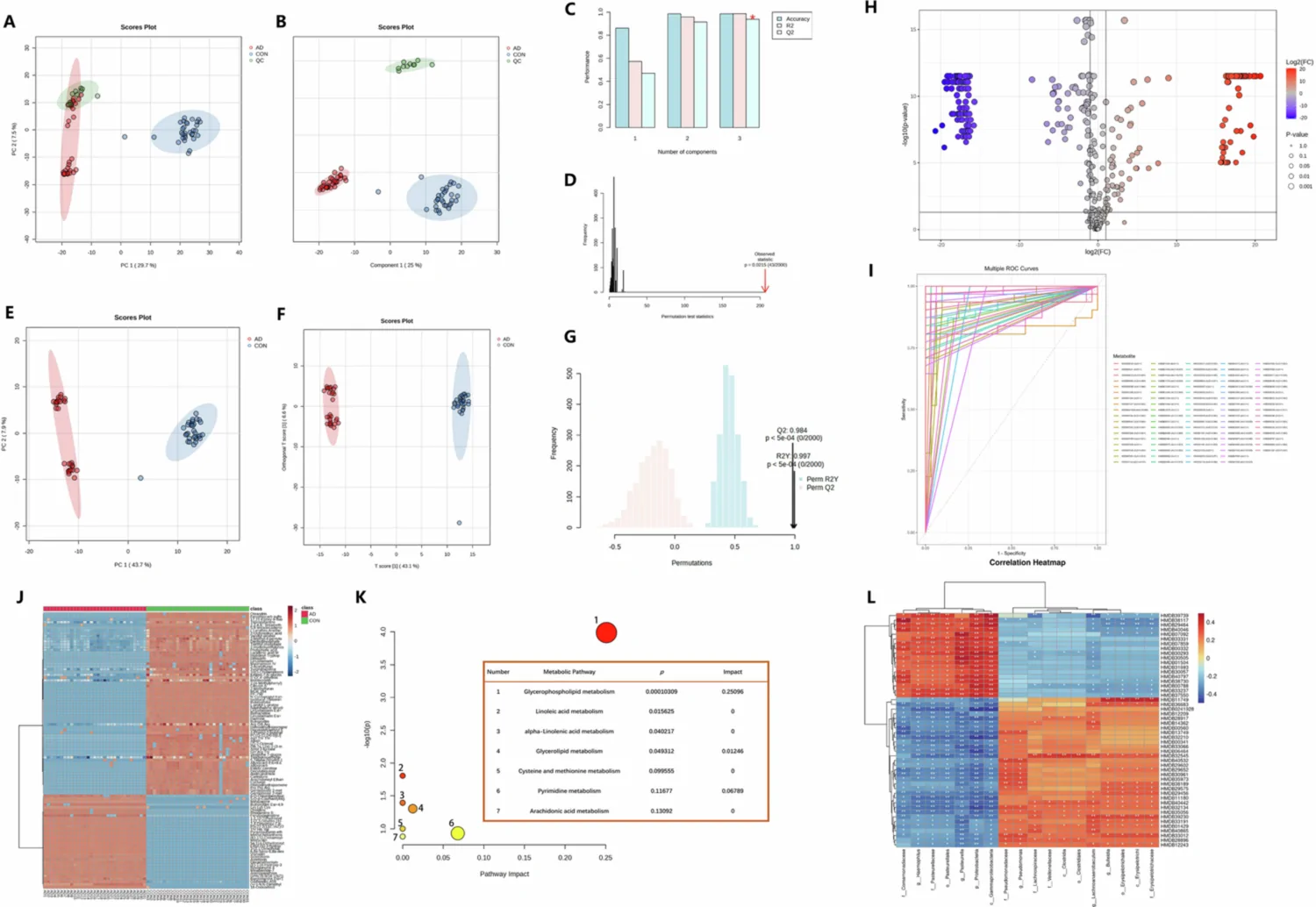
Multivariate and differential metabolite analyses in TB. (A) PCA score plot of the AD, CON, and QC groups. (B) sPLS-DA score plot showing separation among the three groups. (C) Bar plot of model performance metrics (accuracy, R^2^ and Q^2^) for PLS-DA under different numbers of components. (D) Results of a 2,000-time permutation test validating the PLS-DA model. (E) PCA score plot comparing the AD and CON groups. (F) OPLS-DA score plot highlighting group separation. (G) Histogram of R^2^Y and Q^2^ values from the 2,000-time permutation test evaluating the reliability and goodness-of-fit of the OPLS-DA model. (H) Volcano plot showing significantly upregulated and downregulated metabolites (|log₂FC| > 1, –log₁₀(*p*) > 1.3), with FDR-adjusted *p*-values. (I) ROC curve analysis assessing diagnostic performance, with AUC values for classification accuracy. (J) Heatmap of the top 100 altered metabolites, with colour intensity indicating relative abundance. (K) Bubble plot of enriched metabolic pathways, bubble size indicates the number of metabolites, and colour reflects the -log10(*p*) value, where a more intense red colour indicates a higher statistical significance. (L) Spearman correlation analysis between metabolomic and microbiome datasets. Asterisks indicate levels of statistical significance: * denotes *p* < 0.05; ** denotes *p* < 0.01.

#### Screening and analysis of potential biomarkers

After filtering with MPP software, 1,125 metabolites were detected, with 390 putatively identified. Hierarchical clustering of these metabolites showed distinct clustering between groups, as seen in Figure 3J, indicating clear metabolic separation. Differential metabolite analysis (Figure 3H) revealed significant upregulation and downregulation based on the criteria (|log₂FC| > 1, *p* < 0.05). By applying stricter thresholds (VIP > 1, FDR < 0.05), 88 differential metabolites were identified (Appendix Table 1), with mass spectra provided in the appendix. ROC analysis (Figure 3I) showed AUC values ranging from 0.811 to 1.000, with 64 metabolites achieving AUC > 0.90, indicating excellent diagnostic potential. Pathway enrichment analysis (Figure 3K) identified glycerophospholipid metabolism as the most enriched pathway (*p* < 0.001, Impact > 0.25), highlighting its central role in the observed metabolic changes.

#### Spearman correlation analysis

To explore the potential associations between the TB metabolome and microbiome, Spearman correlation analysis was conducted. The results revealed significant correlations between specific metabolites and microbial taxa, suggesting functional interactions between microbial composition and metabolic profiles. As shown in the heatmap (Figure 3L), distinct correlation patterns were observed across metabolite–microbe pairs. For example, the metabolite PA (16:0/18:1(9Z)) (HMDB07859), a glycerophospholipid, exhibited strong positive correlations with several microbial taxa.

### CSF metabolomics data results

As shown in Figure 4A–D, CSF samples from the AD and CON groups were clearly separated by multivariate analysis. The OPLS-DA model showed high reliability and stability, with R^2^Y = 0.992 and Q^2^ = 0.815, and permutation test *p*-values below 0.05, confirming its statistical significance and robustness. Figure 4E presents a volcano plot highlighting the upregulated and downregulated metabolites between the groups. Detailed information on the differential metabolites is provided in the appendix. Figure 4F shows the metabolic pathway enrichment analysis, with glycerophospholipid metabolism significantly enriched (*p* < 0.005) and the highest pathway impact (impact > 0.19), suggesting its central role in metabolic alterations. Cysteine and methionine metabolism also showed strong significance and high pathway impact, indicating its potential involvement in disease-related metabolic disruption.

**Figure 4.**
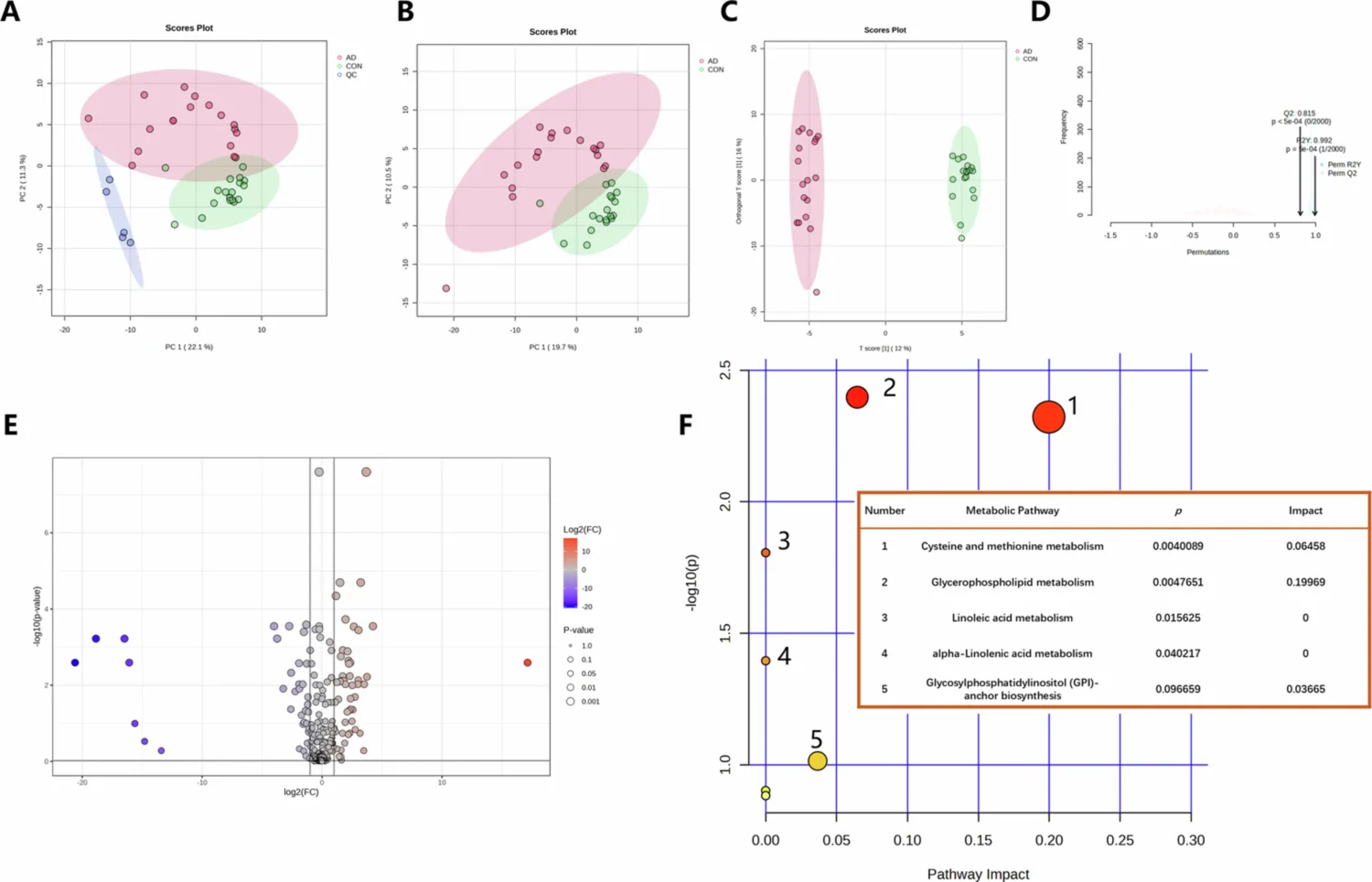
Multivariate and differential metabolite analyses in CSF. (A) PCA score plot showing the overall distribution of AD, CON and QC groups. (B) PCA plot comparing AD and CON groups. (C) OPLS-DA plot showing separation between AD and CON groups. (D) Permutation test (2,000 iterations) evaluating OPLS-DA model fit, with R^2^Y and Q^2^ statistics. (E) Volcano plot highlighting upregulated and downregulated metabolites between AD and CON. (F) Bubble plot of enriched metabolic pathways; bubble size indicates the number of metabolites, and colour reflects the -log10(*p*) value, where a more intense red colour indicates a higher statistical significance.

## Discussion

The TB constitutes a unique ecological niche within the oral cavity. Historically, investigations into TB mainly focused on localized oral lesions and gastrointestinal disorders. In recent years, accumulating evidence has linked TB microbiome alterations to multiple systemic diseases, including rheumatoid arthritis (RA) [40], pneumonia [41], lung malignancies [42], chronic kidney disease (CKD) [43], coronary artery disease (CAD) [43], osteoporosis [44], ischemic stroke [45], and dysmenorrhea [46]. Combined with these published reports and our clinical observations, we targeted the characteristics of TB in patients with AD.

The TCS is a visual scoring system for evaluating tongue coating phenotypes. We first adopted this indicator to preliminarily explore whether TB differs between AD patients and healthy controls. In this study, we enrolled 62 participants who were evenly divided into the AD group and a control group. Significant intergroup differences in TCS were observed, which justified our subsequent multi-omics exploration. To date, existing TB-related research largely relies on tongue image recognition. Although tongue imaging is noninvasive and convenient, it cannot capture subtle microbial and molecular microecological variations. Such invisible alterations are essential for unravelling AD pathogenesis. Therefore, we integrated microbiomics and metabolomics of TB to identify potential diagnostic biomarkers for AD.

Given that hypertension and hyperlipidaemia are common comorbidities among AD patients and that related medications may interfere with oral microecology, we comprehensively collected clinical medication information during subject recruitment. A case–control matching strategy was applied to balance cardiovascular comorbidities between the two cohorts. Demographic analysis confirmed no significant intergroup differences in hypertension and hyperlipidaemia, indicating that the observed microbial and metabolic variations are more likely driven by AD itself rather than concomitant cardiovascular drugs. Although we cannot completely exclude long-term confounding effects of polypharmacy in AD populations, strict exclusion criteria were implemented. Participants with recent antibiotic exposure, periodontal disease, diabetes mellitus or active systemic infection were excluded according to previous evidence linking these conditions to AD and oral dysbiosis. These measures maximized the possibility that the molecular signatures detected in our study reflect pathophysiological changes specific to AD.

Mounting evidence demonstrates that oral microorganisms can enter systemic circulation and further affect peripheral organs and the central nervous system [47]. The bidirectional crosstalk between the oral microbiota and systemic health has been widely reported [27,48–50], and an increasing number of studies have explored its association with AD [51–57]. For instance, microbiota-derived lysophosphatidylcholine can modulate central neuroinflammation and neuronal lipid homeostasis and may alleviate AD pathology via inhibiting ferroptosis [58] (an iron-dependent programmed neuronal cell death pathway strongly implicated in AD neurodegeneration, which can be suppressed by specific microbiota-derived lipid metabolites). Conversely, the oral microbiota has been implicated in AD pathogenesis through the exacerbation of neuroinflammation and the accumulation of amyloid-beta peptides [59]. Additionally, distinct alterations in oral microbial profiles are associated with AD, emphasizing the importance of maintaining good oral hygiene to prevent dysbiosis [60]. Furthermore, periodontitis-related salivary microbiota have been shown to aggravate AD via the gut‒brain axis [61]. Collectively, these findings highlight the critical role of microbes in the pathophysiology of AD.

We first performed 16S rRNA sequencing on TB samples for microbiome analysis. The results revealed distinct differences in microbial composition between the groups. At the phylum level, *Proteobacteria* was the most abundant phylum in the AD group, while *Firmicutes* and *Bacteroidetes* were more predominant in the CON group. Some researchers have reviewed current studies and described the associations between the gut microbiota and biomarker categories involved in the pathogenesis of AD. They highlighted that significant alterations in the gut microbial composition occur across different clinical stages of AD. Notably, *Firmicutes* and *Bacteroidetes* – two dominant phyla in the healthy gut – tend to be downregulated in AD patients [62]. The finding is consistent with the results of our TB analysis. Compared to saliva, which is a more fluid and dynamic sample, the microbial community structure of TB is relatively more stable. Moreover, in contrast to faecal sampling, TB collection is more acceptable to patients and offers a simpler, cleaner, and safer procedure for clinical staff.

In our Q/TOF-MS-based metabolomic analysis, a total of 88 differential metabolites were identified, among which the glycerophospholipid metabolism pathway was significantly enriched, suggesting the potential application of TB samples in AD diagnosis. To further investigate the association between the metabolomics and microbiomics of TB samples, we performed a Spearman correlation analysis, which revealed a significant positive correlation between a specific glycerophospholipid and multiple microbial taxa. This finding is highly consistent with previous studies, which have demonstrated that glycerophospholipids play a crucial role in the interaction between the gut microbiota and neurological diseases. Glycerophospholipids are essential components of cell membranes and play key roles in various physiological processes, including energy storage, signal transduction, cell proliferation, and apoptosis [63]. After the age of 50, most brain lipids gradually decline with ageing [64]. Previous findings have shown that in studies of neurodevelopmental disorders in children, metagenomic and metabolomic analyses revealed enrichment of glycerophospholipid metabolism in the faeces and plasma of patients, which was significantly correlated with specific gut bacterial genera, indicating the potential role of the gut microbiota‒metabolite axis in neurodevelopmental diseases [65]. In the APP/PS1 mouse model, an integrated multi-omics analysis confirmed abnormalities in the gut microbiota-host glycerophospholipid metabolism and found it to be closely associated with neuroinflammatory levels, providing new insights into the role of the gut microbiota in the pathogenesis of AD [66]. Furthermore, research has indicated that icariin can improve glycerophospholipid metabolic disorders in AD mice by regulating the gut microbiota, thereby exerting neuroprotective effects [67]. Similarly, in depression models, the gut microbiota has also been shown to induce depressive-like behaviours via the gut‒brain axis by regulating glycerophospholipid and sphingolipid metabolism, highlighting the importance of the microbiota‒metabolite axis in the pathological process of depression [68,69]. Our results extend these findings and suggest that glycerophospholipids mediate the interaction between oral TB microbiota and AD, deserving further mechanistic investigation.

To further support our findings, we compared our results with the current gold-standard sample type, CSF. Using the same experimental methods and procedures, we analyzed CSF samples and identified a total of 27 differentially abundant metabolites. Notably, pathway analysis revealed that glycerophospholipid metabolism also played a significant role in these CSF samples. This not only lends support to our findings from TB samples but also suggests that the glycerophospholipid metabolism pathway may be of critical importance in AD. Researchers have conducted non-targeted metabolomic studies on the postmortem frontal cortex of AD subjects and found that, compared to control brains, glycerophospholipids – particularly phosphatidylcholine and phosphatidylethanolamine – undergo the most prominent alterations in the AD brain [70]. Other researchers, through the development of novel lipidomics models, have identified distinct alterations in glycerophospholipids in the brains of AD mouse models [71]. Additionally, some researchers have used non-targeted metabolomics and lipidomics approaches to analyze plasma metabolites and identified the glycerophospholipid pathway as a key differential metabolic pathway for distinguishing AD patients from healthy controls [72,73]. These findings underscore the critical role of the glycerophospholipid pathway in Alzheimer's disease. Notably, our non-targeted metabolomic analysis of TB samples also identified this pathway as a significant differential metabolic feature. This not only provides additional evidence supporting its involvement in AD pathophysiology but also highlights the potential of TB as a viable sample type. Compared to other biological samples, such as brain tissue, blood, or CSF, TB offers distinct advantages—being non-invasive, cost-effective, and highly acceptable to patients.

Notably, we originally planned to conduct parallel microbiome detection in both TB and CSF samples. Nevertheless, literature evidence indicates that microorganisms are rarely detectable in CSF from non-infected individuals; microbes only appear in CSF under infectious pathological conditions. Accordingly, only metabolomic profiling was carried out for CSF. This also reflects an important advantage of TB: it enables combined microbiome and metabolome analysis, which cannot be achieved using CSF specimens alone.

## Conclusion

Combining microbiome and non-targeted metabolomics using TB as a sample offers a novel strategy for identifying biomarkers of neurodegenerative diseases. Our findings support TB as a viable specimen for AD detection, showing increased Proteobacteria and decreased Firmicutes and Bacteroidetes in AD. Both TB and CSF samples highlight glycerophospholipid metabolism as a key pathway in AD. TB sampling is non-invasive, more patient-friendly, and easier for community use. With further refinement, it may allow self-sampling for screening, making it promising for future research and clinical use.

However, several limitations of this study should be acknowledged. First, the limited sample size and challenges in metabolomics and microbiome data analysis affect the generalizability of our findings. Standardizing TB collection methods are needed for improved consistency and scientific rigour. Second, this study lacks APOE genotyping data. Given that APOE status, particularly the ε4 allele, is a critical genetic factor associated with lipid metabolism and Alzheimer’s disease, its absence may limit the depth of our analysis. Future studies with larger sample sizes should include APOE stratification to validate these results and explore specific genetic interactions. Third, potential dietary confounding factors cannot be fully eliminated. While patient interviews confirmed no intake of specific supplements, and a pre-sampling fasting protocol was implemented to minimize acute dietary variations, completely ruling out the influence of long-term dietary habits remains challenging, especially in this elderly cohort, who may be unaware of the bioactive compounds in their daily diet. Fourth, the taxonomic resolution of the oral microbiota needs further improvement. The Human Oral Microbiome Database (eHOMD v4.2) offers higher species-level resolution for oral-specific taxa, which would help resolve genus-level ambiguities of oral commensal bacteria. For follow-up studies with expanded cohorts, we will adopt eHOMD for species-level taxonomic annotation to refine the profiling resolution. Despite these limitations, broader adoption of TB sampling could reduce patient burden and infection risk, advancing clinical and community AD research.

## Supplementary Material

Supplementary Material Supplementary_Table_20260803.docx

## Data Availability

Raw data of tongue biofilm metabolomics: https://www.ebi.ac.uk/metabolights/MTBLS12534. Raw data of the tongue biofilm microbiome: PRJNA1268155. Raw data of cerebrospinal fluid metabolomics: http://www.ebi.ac.uk/metabolights/MTBLS12536.

## Appendix

**Table A1.** TC differential metabolite information.

HMDB name	HMDB ID	RT	m/z	Chemical Formula	VIP	FDR < 0.05	log2(FC)	Trend	class	subclass
Macrocarpal I	HMDB41587	4.415	491.2992	C28H42O7	1.139	4.44E-09	2.475	up	Prenol lipids	Sesquiterpenoids
Linusitamarin	HMDB40865	5.514	393.1149	C17H22O9	1.475	8.73E-12	−16.999	down	Organooxygen compounds	Carbohydrates and carbohydrate conjugates
Methyl helianthenoate F glucoside	HMDB40797	4.959	377.1207	C17H22O8	1.521	3.17E-12	17.715	up	Fatty Acyls	Fatty acyl glycosides
Allixin	HMDB40705	15.536	227.1276	C12H18O4	1.386	8.48E-11	−16.480	down	Pyrans	Pyranones and derivatives
Cynaroside A	HMDB40532	4.205	467.1881	C21H32O10	1.143	4.21E-08	−16.815	down	Prenol lipids	Terpene lactones
Dictyoquinazol C	HMDB40442	4.405	343.1296	C18H18N2O5	1.256	2.11E-09	−17.710	down	Diazanaphthalenes	Benzodiazines
1-(2,3-Dihydro-1H-pyrrolizin-5-yl)-1,4-pentanedione	HMDB40046	4.660	206.1176	C12H15NO2	1.521	3.17E-12	16.675	up	Pyrrolizines	–
5-Hydroxy-7-methoxy-2-tritriacontyl-4H-1-benzopyran-4-one	HMDB39739	17.104	655.5658	C43H74O4	1.024	1.49E-05	1.814	up	Benzopyrans	1-benzopyrans
Citrusin A	HMDB39230	8.667	539.2100	C26H34O12	1.520	3.17E-12	−17.719	down	Lignan glycosides	–
(7'R)-(+)-Lyoniresinol 9'-glucoside	HMDB38922	4.335	600.2635	C28H38O13	1.255	2.11E-09	−17.247	down	Lignan glycosides	–
5-O-p-Coumaroylnigrumin	HMDB38730	10.050	439.1727	C20H23NO9	1.441	5.82E-12	6.303	up	Tannins	–
Acidissiminin epoxide	HMDB38620	17.141	676.4976	C43H65NO5	1.155	1.61E-08	4.087	up	Benzene and substituted derivatives	Benzoic acids and derivatives
Carindone	HMDB38189	16.200	530.3467	C31H44O6	1.474	8.73E-12	−18.509	down	Prenol lipids	Sesquiterpenoids
6-O-Oleuropeoylsucrose	HMDB38117	16.169	526.2493	C22H36O13	1.426	2.72E-11	17.844	up	Saccharolipids	–
2-Methyl-4-pentyloxazole	HMDB37856	5.830	171.1492	C9H15NO	1.492	2.09E-16	−1.587	down	Azoles	Oxazoles
Ethyl vanillin isobutyrate	HMDB37683	4.801	237.1112	C13H16O4	1.248	2.11E-09	−17.851	down	Phenol esters	–
Sandoricin	HMDB37555	11.292	611.2458	C31H40O11	1.053	2.84E-07	15.932	up	Carboxylic acids and derivatives	Tetracarboxylic acids and derivatives
2,4-Dihydroxy-7,8-dimethoxy-2H-1,4-benzoxazin-3(4H)-one 2-glucoside	HMDB37550	4.998	421.1470	C16H21NO11	1.521	3.17E-12	16.576	up	Organooxygen compounds	Carbohydrates and carbohydrate conjugates
1,2,3-Tris(1-ethoxyethoxy)propane	HMDB37162	5.263	309.2266	C15H32O6	1.105	1.10E-07	−17.212	down	Glycerolipids	–
5,7alpha-Dihydro-1,4,4,7a-tetramethyl-4H-indene	HMDB36683	16.903	175.1479	C13H18	1.338	6.27E-11	−3.836	down	Unsaturated hydrocarbons	Branched unsaturated hydrocarbons
Lucidenic acid M	HMDB35973	15.486	463.3015	C27H42O6	1.429	2.72E-11	−17.406	down	Prenol lipids	Triterpenoids
1-(3-Hydroxy-4-methoxyphenyl)-1,2-ethanediol	HMDB35056	4.146	185.0805	C9H12O4	1.217	5.95E-09	−17.275	down	Phenols	Methoxyphenols
Gibberellin A59	HMDB35051	7.276	383.1106	C19H20O7	1.515	2.09E-16	3.359	up	Prenol lipids	Diterpenoids
Lycoperoside D	HMDB34484	4.426	740.4614	C39H65NO12	1.137	4.21E-08	−16.250	down	Steroids and steroid derivatives	Steroidal glycosides
(E)-1-[4-Hydroxy-3-(3-methyl-1,3-butadienyl)phenyl]-2-(3,5-dihydroxyphenyl)ethylene	HMDB33331	14.609	317.1154	C19H18O3	1.520	3.17E-12	17.638	up	Stilbenes	–
1,2,10-Trihydroxydihydro-trans-linalyl oxide 7-O-beta-D-glucopyranoside	HMDB33237	4.993	400.2170	C16H30O10	1.521	3.17E-12	17.980	up	Organooxygen compounds	Carbohydrates and carbohydrate conjugates
2,2,4,4,-Tetramethyl-6-(1-oxopropyl)-1,3,5-cyclohexanetrione	HMDB33191	7.422	261.1095	C13H18O4	1.444	5.82E-12	−5.815	down	Prenol lipids	Monoterpenoids
2-Acetylfuran	HMDB33127	7.256	111.0439	C6H6O2	1.346	2.64E-10	−15.729	down	Organooxygen compounds	Carbonyl compounds
(-)-erythro-Anethole glycol 1-glucoside	HMDB33066	7.278	345.1543	C16H24O8	1.508	5.36E-11	−1.858	down	Organooxygen compounds	Carbohydrates and carbohydrate conjugates
Vestitone 7-glucoside	HMDB33012	5.015	435.1634	C22H26O9	1.385	8.48E-11	−17.951	down	Isoflavonoids	Isoflavonoid O-glycosides
3-(1,1-Dimethylallyl)scopoletin 7-glucoside	HMDB32853	5.077	445.1467	C21H26O9	1.520	3.17E-12	16.317	up	Coumarins and derivatives	Coumarin glycosides
Tripropylamine	HMDB32545	4.425	144.1746	C9H21N	1.439	5.36E-11	−2.444	down	Organonitrogen compounds	Amines
(+/-)-N,N-Dimethyl menthyl succinamide	HMDB32236	16.179	186.2215	C12H24	1.507	3.17E-12	20.071	up	Unsaturated hydrocarbons	Branched unsaturated hydrocarbons
N-Cyclopropyl-trans-2-cis-6-nonadienamide	HMDB32210	7.760	211.1789	C12H19NO	1.521	3.17E-12	−16.623	down	Fatty Acyls	Fatty amides
Gentiobiosyl 2-methyl-6-oxo-2E,4E-heptadienoate	HMDB32134	4.120	501.1574	C20H30O13	1.470	8.73E-12	−19.068	down	Organooxygen compounds	Carbohydrates and carbohydrate conjugates
2,4,12-Octadecatrienoic acid isobutylamide	HMDB32033	14.921	334.3105	C22H39NO	1.158	1.61E-08	19.394	up	Fatty Acyls	Fatty amides
Ethyl (S)-3-hydroxybutyrate glucoside	HMDB31693	4.920	295.1388	C12H22O8	1.521	3.17E-12	18.057	up	Fatty Acyls	Fatty acyl glycosides
Homodihydrojasmone	HMDB31181	6.120	181.1585	C12H20O	1.426	2.15E-11	−5.463	down	Organooxygen compounds	Carbonyl compounds
1-Pentadecene	HMDB31082	9.605	228.2685	C15H30	1.255	3.17E-12	−1.221	down	Unsaturated hydrocarbons	Unsaturated aliphatic hydrocarbons
2-Pentadecanone	HMDB31081	9.173	244.2633	C15H30O	1.127	1.31E-08	−5.096	down	Organooxygen compounds	Carbonyl compounds
2-Dodecylbenzenesulfonic acid	HMDB31031	4.119	327.2013	C18H30O3S	1.100	1.17E-09	−1.759	down	Benzene and substituted derivatives	Benzenesulfonic acids and derivatives
2-Octenal	HMDB30961	4.987	127.1116	C8H14O	1.432	2.72E−11	−18.643	down	Organooxygen compounds	Carbonyl compounds
Janthitrem B	HMDB30528	4.352	586.3512	C37H47NO5	1.122	3.77E-08	−5.757	down	Naphthopyrans	–
Bargustanine	HMDB30505	14.104	523.2457	C29H34N2O7	1.517	3.17E-12	18.362	up	Isoquinolines and derivatives	Benzylisoquinolines
(3R,5Z)-5-Octene-1,3-diol	HMDB30368	15.128	145.1224	C8H16O2	1.266	2.11E-09	−16.364	down	Fatty Acyls	Fatty alcohols
Platydesminium	HMDB30342	6.814	292.1807	C16H20NO3	1.059	2.84E-07	16.847	up	Quinolines and derivatives	Dihydrofuranoquinolines
(E)-1-O-Cinnamoyl-beta-D-glucose	HMDB30293	4.919	333.0945	C15H18O7	1.521	3.17E-12	18.177	up	Cinnamic acids and derivatives	Cinnamic acid esters
Boviquinone 4	HMDB30057	15.537	435.2506	C26H36O4	1.520	3.17E-12	17.379	up	Prenol lipids	Diterpenoids
Momordol	HMDB29804	15.485	463.3378	C26H48O5	1.176	1.61E-08	-16.730	down	Fatty Acyls	Fatty alcohols
Montecristin	HMDB29795	17.076	575.5020	C37H66O4	1.310	6.08E-16	−1.527	down	Fatty Acyls	Fatty alcohols
9-Pentadecenoic acid	HMDB29765	13.292	258.2454	C15H28O2	1.132	4.21E-08	−16.489	down	Fatty Acyls	Fatty acids and conjugates
2,3,5-Trimethylfuran	HMDB29721	16.160	111.0806	C7H10O	1.244	8.72E-10	−5.069	down	Heteroaromatic compounds	–
2,6-Dimethoxy-4-methylphenol	HMDB29680	16.179	169.0856	C9H12O3	1.295	7.67E-10	−17.073	down	Phenols	Methoxyphenols
2-(4-Methylphenyl)-2-propanol	HMDB29652	5.024	151.1117	C10H14O	1.520	3.17E-12	−18.820	down	Benzene and substituted derivatives	Phenylpropanes
4-Phenyl-2-butenal	HMDB29602	16.174	147.0802	C10H10O	1.392	1.89E-11	−5.020	down	Benzene and substituted derivatives	–
(+)-cis-5,6-Dihydro-5-hydroxy-4-methoxy-6-(2-phenylethyl)-2H-pyran-2-one	HMDB29575	4.393	249.1119	C14H16O4	1.249	2.11E-09	−18.377	down	Kavalactones	–
Asteltoxin	HMDB29464	16.064	441.1883	C23H30O7	1.520	3.17E-12	18.701	up	Furofurans	–
Amyl 2-furoate	HMDB29456	4.546	183.1015	C10H14O3	1.429	2.72E-11	−19.266	down	Furans	Furoic acid and derivatives
Lysyltyrosine	HMDB28963	3.181	310.1777	C15H23N3O4	1.087	1.10E-07	−16.831	down	Carboxylic acids and derivatives	Amino acids, peptides, and analogues
Isoleucyl-Threonine	HMDB28917	7.695	250.1775	C10H20N2O4	1.521	3.17E-12	−17.832	down	Carboxylic acids and derivatives	Amino acids, peptides, and analogues
Histidyltryptophan	HMDB28896	9.370	359.1824	C17H19N5O3	1.429	2.72E-11	−17.668	down	Carboxylic acids and derivatives	Amino acids, peptides, and analogues
Decamethonium	HMDB15375	13.655	281.2947	C16H38N2	1.520	3.17E-12	18.030	up	Organonitrogen compounds	Quaternary ammonium salts
Bethanidine	HMDB14362	4.142	178.1336	C10H15N3	1.521	3.17E-12	−18.181	down	Benzene and substituted derivatives	
2-Methylfuran	HMDB13749	7.402	105.0334	C5H6O	1.521	3.17E-12	−16.467	down	Heteroaromatic compounds	–
Kinetin-7-N-glucoside	HMDB12243	8.660	395.1675	C16H19N5O6	1.423	3.34E-16	−1.444	down	Organooxygen compounds	Carbohydrates and carbohydrate conjugates
Diethylphosphate	HMDB12209	5.674	155.0470	C4H11O4P	1.486	2.09E-16	−1.157	down	Organic phosphoric acids and derivatives	Phosphate esters
Δ-valerolactam	HMDB11749	3.319	100.0758	C5H9NO	1.113	4.21E-08	−20.699	down	Piperidines	Piperidinones
MG(0:0/15:0/0:0)	HMDB11532	8.010	334.2944	C18H36O4	1.027	1.15E-07	2.682	up	Glycerolipids	Monoradylglycerols
PE(P-16:0/16:1(9Z))	HMDB11339	16.988	674.5102	C37H72NO7P	1.192	2.26E-08	−3.857	down	Glycerophospholipids	Glycerophosphoethanolamines
Prolylproline	HMDB11180	17.393	213.1216	C10H16N2O3	1.521	3.17E-12	−16.339	down	Carboxylic acids and derivatives	Amino acids, peptides, and analogues
DG(O-16:0/18:0/0:0)	HMDB11146	16.973	605.5472	C37H74O4	1.229	8.20E-10	−3.870	down	Glycerolipids	Diradylglycerols
PC(15:0/22:2(13Z,16Z))	HMDB07954	17.101	800.6217	C45H86NO8P	1.233	6.01E-09	2.904	up	Glycerophospholipids	Glycerophosphocholines
PC(15:0/18:1(11Z))	HMDB07938	17.114	747.5763	C41H80NO8P	1.149	1.27E-08	−4.612	down	Glycerophospholipids	Glycerophosphocholines
PA(16:0/18:1(9Z))	HMDB07859	17.200	675.4962	C37H71O8P	1.520	3.17E-12	19.256	up	Glycerophospholipids	Glycerophosphates
DG(20:3(5Z,8Z,11Z)/22:6(4Z,7Z,10Z,13Z,16Z,19Z)/0:0)	HMDB07469	13.102	681.5294	C45H70O5	1.166	1.61E-08	−19.832	down	Glycerolipids	Diradylglycerols
DG(18:1(11Z)/22:5(4Z,7Z,10Z,13Z,16Z)/0:0)	HMDB07206	13.098	691.5297	C43H72O5	1.032	2.78E-07	−6.350	down	Glycerolipids	Diradylglycerols
DG(15:0/22:6(4Z,7Z,10Z,13Z,16Z,19Z)/0:0)	HMDB07092	17.215	649.4779	C40H66O5	1.259	3.42E-10	5.625	up	Glycerolipids	Diradylglycerols
Elaidic carnitine	HMDB06464	9.673	449.3478	C25H47NO4	1.473	8.73E-12	−17.368	down	Fatty Acyls	Fatty acid esters
3,4-Dihydroxyphenylethanol	HMDB05784	5.011	155.0706	C8H10O3	1.219	5.95E-09	−17.892	down	Phenols	Tyrosols and derivatives
N-Palmitoyl Leucine	HMDB0241928	16.975	370.3304	C22H43NO3	1.159	5.16E-08	−4.295	down	Carboxylic acids and derivatives	Amino acids, peptides, and analogues
3-Methylthiopropionic acid	HMDB01527	14.622	121.0293	C4H8O2S	1.234	9.15E-06	1.861	up	Fatty Acyls	Fatty acids and conjugates
Aminoparathion	HMDB01504	7.795	279.0933	C10H16NO3PS	1.520	3.17E-12	18.046	up	Organic thiophosphoric acids and derivatives	Thiophosphoric acid esters
Phosphate	HMDB01429	5.675	98.9845	H3O4P	1.500	2.09E-16	−1.220	down	Non-metal oxoanionic compounds	Non-metal phosphates
Orotidine	HMDB00788	4.844	289.0682	C10H12N2O8	1.474	8.73E-12	16.572	up	Pyrimidine nucleosides	–
5,8-Tetradecadienoic acid	HMDB00560	17.375	247.1650	C14H24O2	1.431	2.72E-11	−16.503	down	Fatty Acyls	Fatty acids and conjugates
5-Androstene-3b,16b,17a-triol	HMDB00523	11.895	307.2264	C19H30O3	1.138	4.21E-08	−16.596	down	Steroids and steroid derivatives	Androstane steroids
2-Octenedioic acid	HMDB00341	7.277	367.1365	C8H12O4	1.516	2.09E-16	−2.673	down	Fatty Acyls	Fatty acids and conjugates
18-Oxocortisol	HMDB00332	14.476	399.1790	C21H28O6	1.492	4.43E-12	8.973	up	Steroids and steroid derivatives	Hydroxysteroids

## References

[1] Porsteinsson AP , Isaacson RS , Knox S , et al. Diagnosis of early alzheimer’s disease: clinical practice in 2021. J Prev Alzheimers Dis. 2021;8(3):371–386. doi: 10.14283/jpad.2021.23 PMC12280795 34101796

[2] Graff-Radford J , Yong KXX , Apostolova LG , et al. New insights into atypical alzheimer’s disease in the era of biomarkers. Lancet Neurol. 2021;20(3):222–234. doi: 10.1016/S1474-4422(20)30440-3 PMC8056394 33609479

[3] Scheltens P , De Strooper B , Kivipelto M , et al. Alzheimer’s disease. Lancet. 2021;397(10284):1577–1590. doi: 10.1016/S0140-6736(20)32205-4 PMC8354300 33667416

[4] Ph N , P D . Status of alzheimer’s and parkinson’s disease detections and treatments. ACS Chem Neurosci. 2026;17(13). doi: 10.1021/acschemneuro.6c00096 42313891

[5] Jain SS , Ang TFA , Sunderaraman P , et al. Sex differences in informal caregiving costs and burden in alzheimer disease: a multinational cross-sectional analysis. BMC Public Health. 2026;26(1):1938. doi: 10.1186/s12889-026-27524-9 PMC13285181 42063002

[6] Zhang XX , Tian Y , Wang ZT , et al. The epidemiology of alzheimer’s disease modifiable risk factors and prevention. J Prev Alzheimers Dis. 2021;8(3):313–321. doi: 10.14283/jpad.2021.15 PMC12280729 34101789

[7] Danser MM , Gómez SM , Van der Weijden GA . Tongue coating and tongue brushing: a literature review. Int J Dent Hyg. 2003;1(3):151–158. doi: 10.1034/j.1601-5037.2003.00034.x 16451515

[8] Seerangaiyan K , Jüch F , Winkel EG . Tongue coating: its characteristics and role in intra-oral halitosis and general health-a review. J Breath Res. 2018;12(3):034001. doi: 10.1088/1752-7163/aaa3a1 29269592

[9] Van Tornout M , Dadamio J , Coucke W , et al. Tongue coating: related factors. J Clin Periodontol. 2013;40(2):180–185. doi: 10.1111/jcpe.12031 23278504

[10] Kim J , Son J , Jang S , et al. Availability of tongue diagnosis system for assessing tongue coating thickness in patients with functional dyspepsia. Evid-Based Complement Altern Med ECAM. 2013;2013:348272. doi: 10.1155/2013/348272 PMC3789501 24159343

[11] Segawa M , Iizuka N , Ogihara H , et al. Construction of a standardized tongue image database for diagnostic education: development of a tongue diagnosis e-Learning system. Front Med Technol. 2021;3:760542. doi: 10.3389/fmedt.2021.760542 PMC8757883 35047962

[12] Yuan L , Yang L , Zhang S , et al. Development of a tongue image-based machine learning tool for the diagnosis of gastric cancer: a prospective multicentre clinical cohort study. EClinicalMedicine. 2023;57:101834. doi: 10.1016/j.eclinm.2023.101834 PMC9941057 36825238

[13] Park S , Lee ES , Kim A , et al. Development of a novel tongue biofilm index using bacterial biofluorescence. Sci Rep. 2024;14(1):30196. doi: 10.1038/s41598-024-80696-5 PMC11618654 39633009

[14] Zhang Y , Lo KL , Liman AN , et al. Tongue-coating microbial and metabolic characteristics in halitosis. J Dent Res. 2024;103(5):484–493. doi: 10.1177/00220345241230067 38623900

[15] Mu X , Ji C , Wang Q , et al. Non-targeted metabolomics reveals diagnostic biomarker in the tongue coating of patients with chronic gastritis. J Pharm Biomed Anal. 2019;174:541–551. doi: 10.1016/j.jpba.2019.06.025 PMC7383221 31255854

[16] Cui J , Cui H , Yang M , et al. Tongue coating microbiome as a potential biomarker for gastritis including precancerous cascade. Protein Cell. 2019;10(7):496–509. doi: 10.1007/s13238-018-0596-6 PMC6588651 30478535

[17] Guo XJ , Jiang T , Ma XX , et al. Relationships between diurnal changes of tongue coating microbiota and intestinal microbiota. Front Cell Infect Microbiol. 2022;12:813790. doi: 10.3389/fcimb.2022.813790 PMC9008461 35433494

[18] Mu X , Ji C , Wang Q , et al. Non-targeted metabolomics reveals diagnostic biomarker in the tongue coating of patients with chronic gastritis. J Pharm Biomed Anal. 2019;174:541–551. doi: 10.1016/j.jpba.2019.06.025 PMC7383221 31255854

[19] Kapila YL . Oral health’s inextricable connection to systemic health: special populations bring to bear multimodal relationships and factors connecting periodontal disease to systemic diseases and conditions. Periodontol 2000. 2021;87(1):11–16. doi: 10.1111/prd.12398 PMC8457130 34463994

[20] Pathak JL , Yan Y , Zhang Q , et al. The role of oral microbiome in respiratory health and diseases. Respir Med. 2021;185:106475. doi: 10.1016/j.rmed.2021.106475 34049183

[21] Li Y , Cui J , Liu Y , et al. Oral, tongue-coating microbiota, and metabolic disorders: a novel area of interactive research. Front Cardiovasc Med. 2021;8. doi: 10.3389/fcvm.2021.730203 PMC8417575 34490384

[22] Ali Mohammed MM , Al Kawas S , Al-Qadhi G . Tongue-coating microbiome as a cancer predictor: a scoping review. Arch Oral Biol. 2021;132:105271. doi: 10.1016/j.archoralbio.2021.105271 34610507

[23] Zhu G , Zhao J , Zhang H , et al. Gut microbiota and its metabolites: bridge of dietary nutrients and alzheimer’s disease. Adv Nutr Bethesda Md. 2023;14(4):819–839. doi: 10.1016/j.advnut.2023.04.005 PMC10334159 37075947

[24] Chandra S , Vassar RJ . Gut microbiome-derived metabolites in alzheimer’s disease: regulation of immunity and potential for therapeutics. Immunol Rev. 2024;327(1):33–42. doi: 10.1111/imr.13412 PMC12309846 39440834

[25] Zhao H , Zhou X , Song Y , et al. Multi-omics analyses identify gut microbiota-fecal metabolites-brain-cognition pathways in the alzheimer’s disease continuum. Alzheimers Res Ther. 2025;17(1):36. doi: 10.1186/s13195-025-01683-0 PMC11786436 39893498

[26] Chen Y , Li Y , Fan Y , et al. Gut microbiota-driven metabolic alterations reveal gut-brain communication in alzheimer’s disease model mice. Gut Microbes. 2024;16(1):2302310. doi: 10.1080/19490976.2024.2302310 PMC10807476 38261437

[27] Grevich S , Lee P , Leroux B , et al. Oral health and plaque microbial profile in juvenile idiopathic arthritis. Pediatr Rheumatol Online J. 2019;17(1):81. doi: 10.1186/s12969-019-0387-5 PMC6916162 31842923

[28] Cao D , Zhang Y , Zhang S , et al. Risk of alzheimer’s disease and genetically predicted levels of 1400 plasma metabolites: a mendelian randomization study. Sci Rep. 2024;14(1):26078. doi: 10.1038/s41598-024-77921-6 PMC11525545 39478193

[29] Jung YH , Chae CW , Han HJ . The potential role of gut microbiota-derived metabolites as regulators of metabolic syndrome-associated mitochondrial and endolysosomal dysfunction in alzheimer’s disease. Exp Mol Med. 2024;56(8):1691–1702. doi: 10.1038/s12276-024-01282-3 PMC11372123 39085351

[30] Zhang E , Dai F , Tao L , et al. Immune cells: mediators in the metabolites and alzheimer’s disease. J Alzheimers Dis JAD. 2025;103(4):1277–1288. doi: 10.1177/13872877241313140 39876754

[31] Jacob M , Lopata AL , Dasouki M , et al. Metabolomics toward personalized Medicine. Mass Spectrom Rev. 2019;38(3):221–238. doi: 10.1002/mas.21548 29073341

[32] Cui L , Lu H , Lee YH . Challenges and emergent solutions for LC-MS/MS based untargeted metabolomics in diseases. Mass Spectrom Rev. 2018;37(6):772–792. doi: 10.1002/mas.21562 29486047

[33] Yang L , Xiang Z , Zou J , et al. Comprehensive analysis of the relationships between the gut microbiota and fecal metabolome in individuals with primary sjogren’s syndrome by 16S rRNA sequencing and LC-MS-Based metabolomics. Front Immunol. 2022;13:874021. doi: 10.3389/fimmu.2022.874021 PMC9130595 35634334

[34] Liu J , Geng W , Sun H , et al. Integrative metabolomic characterisation identifies altered portal vein serum metabolome contributing to human hepatocellular carcinoma. Gut. 2022;71(6):1203–1213. doi: 10.1136/gutjnl-2021-325189 PMC9120406 34344785

[35] Zhao F , An R , Wang L , et al. Specific gut microbiome and serum metabolome changes in lung cancer patients. Front Cell Infect Microbiol. 2021;11:725284. doi: 10.3389/fcimb.2021.725284 PMC8435782 34527604

[36] Shao Y , Li T , Liu Z , et al. Comprehensive metabolic profiling of parkinson’s disease by liquid chromatography-mass spectrometry. Mol Neurodegener. 2021;16(1):4. doi: 10.1186/s13024-021-00425-8 PMC7825156 33485385

[37] Curry KD , Wang Q , Nute MG , et al. Emu: species-level microbial community profiling of full-length 16S rRNA Oxford nanopore sequencing data. Nat Methods. 2022;19(7):845–853. doi: 10.1038/s41592-022-01520-4-+ PMC9939874 35773532

[38] Hassler HB , Probert B , Moore C , et al. Phylogenies of the 16S rRNA gene and its hypervariable regions lack concordance with core genome phylogenies. Microbiome. 2022;10(1):104. doi: 10.1186/s40168-022-01295-y PMC9264627 35799218

[39] Aimetti M , Perotto S , Castiglione A , et al. Prevalence estimation of halitosis and its association with oral health-related parameters in an adult population of a city in north Italy. J Clin Periodontol. 2015;42(12):1105–1114. doi: 10.1111/jcpe.12474 26477536

[40] Kroese JM , Brandt BW , Buijs MJ , et al. Differences in the oral microbiome in patients with early rheumatoid arthritis and individuals at risk of rheumatoid arthritis compared to healthy individuals. Arthritis Rheumatol. 2021;73(11):1986–1993. doi: 10.1002/art.41780 PMC8596438 33949151

[41] Takeshita T , Tomioka M , Shimazaki Y , et al. Microfloral characterization of the tongue coating and associated risk for pneumonia-related health problems in institutionalized older adults. J Am Geriatr Soc. 2010;58(6):1050–1057. doi: 10.1111/j.1532-5415.2010.02867.x 20487070

[42] Su W , Xu ZY , Wang ZQ , et al. Objectified study on tongue images of patients with lung cancer of different syndromes. Chin J Integr Med. 2011;17(4):272–276. doi: 10.1007/s11655-011-0702-6 21509670

[43] Hao Y , Yuan X , Yan J , et al. Metabolomic markers in tongue-coating samples from damp phlegm pattern patients of coronary heart disease and chronic renal failure. Dis Markers. 2019;2019:4106293. doi: 10.1155/2019/4106293 PMC6754907 31583028

[44] Pereira IF , Brasileiro CB , Kleperon NP , et al. Comparative study of oral and salivary parameters in patients with and without loss of bone mass. Braz Oral Res. 2018;32:e54. doi: 10.1590/1807-3107bor-2018.vol32.0054 29898022

[45] Huang YS , Wu HK , Chang HH , et al. Exploring the pivotal variables of tongue diagnosis between patients with acute ischemic stroke and health participants. J Tradit Complement Med. 2022;12(5):505–510. doi: 10.1016/j.jtcme.2022.04.001 PMC9446173 36081819

[46] Kim J , Lee H , Kim H , et al. Differences in the tongue features of primary dysmenorrhea patients and controls over a normal menstrual cycle. Evid-Based Complement Altern Med ECAM. 2017;2017:6435702. doi: 10.1155/2017/6435702 PMC5469986 28642801

[47] Wan J , Fan H . Oral microbiome and alzheimer’s disease. Microorganisms. 2023;11(10):10. doi: 10.3390/microorganisms11102550 PMC10609607 37894208

[48] Pathak JL , Yan Y , Zhang Q , et al. The role of oral microbiome in respiratory health and diseases. Respir Med. 2021;185:106475. doi: 10.1016/j.rmed.2021.106475 34049183

[49] Santacroce L , Passarelli PC , Azzolino D , et al. Oral microbiota in human health and disease: a perspective. Exp Biol Med Maywood NJ. 2023;248(15):1288–1301. doi: 10.1177/15353702231187645 PMC10625343 37688509

[50] Yamashita Y , Takeshita T . The oral microbiome and human health. J Oral Sci. 2017;59(2):201–206. doi: 10.2334/josnusd.16-0856 28637979

[51] Jungbauer G , Stähli A , Zhu X , et al. Periodontal microorganisms and alzheimer disease - A causative relationship?. Periodontol 2000. 2022;89(1):59–82. doi: 10.1111/prd.12429 PMC9314828 35244967

[52] Dibello V , Lozupone M , Manfredini D , et al. Oral frailty and neurodegeneration in alzheimer’s disease. Neural Regen Res. 2021;16(11):2149–2153. doi: 10.4103/1673-5374.310672 PMC8354109 33818486

[53] Liu S , Dashper SG , Zhao R . Association between oral bacteria and alzheimer’s disease: a systematic review and meta-analysis. J Alzheimers Dis JAD. 2023;91(1):129–150. doi: 10.3233/JAD-220627 36404545

[54] Borsa L , Dubois M , Sacco G , et al. Analysis the link between periodontal diseases and alzheimer’s disease: a systematic review. Int J Environ Res Public Health. 2021;18(17):9312. doi: 10.3390/ijerph18179312 PMC8430572 34501899

[55] Popescu C , Munteanu C , Anghelescu A , et al. Novelties on neuroinflammation in alzheimer’s disease-focus on gut and oral microbiota involvement. Int J Mol Sci. 2024;25(20):11272. doi: 10.3390/ijms252011272 PMC11508522 39457054

[56] Aguayo S , Schuh CMAP , Vicente B , et al. Association between alzheimer’s disease and oral and gut microbiota: are pore forming proteins the missing link?. J Alzheimers Dis JAD. 2018;65(1):29–46. doi: 10.3233/JAD-180319 30040725

[57] Fox M , Knorr DA , Haptonstall KM . Alzheimer’s disease and symbiotic microbiota: an evolutionary Medicine perspective. Ann N Y Acad Sci. 2019;1449(1):3–24. doi: 10.1111/nyas.14129 PMC9495288 31180143

[58] Zha X , Liu X , Wei M , et al. Microbiota-derived lysophosphatidylcholine alleviates alzheimer’s disease pathology via suppressing ferroptosis. Cell Metab. 2025;37(1):169–186.e9. doi: 10.1016/j.cmet.2024.10.006 39510074

[59] Bello-Corral L , Alves-Gomes L , Fernández-Fernández JA , et al. Implications of gut and oral microbiota in neuroinflammatory responses in alzheimer’s disease. Life Sci. 2023;333:122132. doi: 10.1016/j.lfs.2023.122132 37793482

[60] Sureda A , Daglia M , Argüelles Castilla S , et al. Oral microbiota and alzheimer’s disease: do all roads lead to Rome?. Pharmacol Res. 2020;151:104582. doi: 10.1016/j.phrs.2019.104582 31794871

[61] Lu J , Zhang S , Huang Y , et al. Periodontitis-related salivary microbiota aggravates alzheimer’s disease via gut-brain axis crosstalk. Gut Microbes. 2022;14(1):2126272. doi: 10.1080/19490976.2022.2126272 PMC9542625 36175166

[62] Liang Y , Liu C , Cheng M , et al. The link between gut microbiome and alzheimer’s disease: from the perspective of new revised criteria for diagnosis and staging of alzheimer’s disease. Alzheimers Dement. 2024;20(8):5771–5788. doi: 10.1002/alz.14057 PMC11350031 38940631

[63] Petrenko V , Sinturel F , Riezman H , et al. Lipid metabolism around the body clocks. Prog Lipid Res. 2023;91:101235. doi: 10.1016/j.plipres.2023.101235 37187314

[64] Kosicek M , Hecimovic S . Phospholipids and alzheimer’s disease: alterations, mechanisms and potential biomarkers. Int J Mol Sci. 2013;14(1):1310–1322. doi: 10.3390/ijms14011310 PMC3565322 23306153

[65] Wang Q , Luo Y , Mao C , et al. Combined metagenomic and metabolomic analyses reveal gut microbiota dysbiosis and metabolic dysfunction in pediatric neurodevelopmental disorders. Front Immunol. 2025;16:1645137. doi: 10.3389/fimmu.2025.1645137 PMC12436362 40963595

[66] Qian X , Hai W , Chen S , et al. Multi-omics data reveals aberrant gut microbiota-host glycerophospholipid metabolism in association with neuroinflammation in APP/PS1 mice. Gut Microbes. 2023;15(2):2282790. doi: 10.1080/19490976.2023.2282790 PMC10730179 37992400

[67] Liu Y , Li H , Wang X , et al. Anti-alzheimers molecular mechanism of icariin: insights from gut microbiota, metabolomics, and network pharmacology. J Transl Med. 2023;21(1):277. doi: 10.1186/s12967-023-04137-z PMC10124026 37095548

[68] Tian T , Mao Q , Xie J , et al. Multi-omics data reveals the disturbance of glycerophospholipid metabolism caused by disordered gut microbiota in depressed mice. J Adv Res. 2022;39:135–145. doi: 10.1016/j.jare.2021.10.002 PMC9263645 35777903

[69] Cao Y , Fan X , Zang T , et al. Gut microbiota causes depressive phenotype by modulating glycerophospholipid and sphingolipid metabolism via the gut-brain axis. Psychiatry Res. 2025;346:116392. doi: 10.1016/j.psychres.2025.116392 39933221

[70] Paglia G , Stocchero M , Cacciatore S , et al. Unbiased metabolomic investigation of alzheimer’s disease brain points to dysregulation of mitochondrial aspartate metabolism. J Proteome Res. 2016;15(2):608–618. doi: 10.1021/acs.jproteome.5b01020 PMC5751881 26717242

[71] Xu S , Zhu Z , Delafield DG , et al. Spatially and temporally probing distinctive glycerophospholipid alterations in alzheimer’s disease mouse brain via high-resolution ion mobility-enabled sn-position resolved lipidomics. Nat Commun. 2024;15(1):6252. doi: 10.1038/s41467-024-50299-9 PMC11269705 39048572

[72] Vardarajan B , Kalia V , Manly J , et al. Differences in plasma metabolites related to alzheimer’s disease, *apoe* ε4 status, and ethnicity. Alzheimers Dement-Transl Res Clin Interv. 2020;6(1):e12025. doi: 10.1002/trc2.12025 PMC7201178 32377558

[73] Zhang L , Li L , Meng F , et al. Serum metabolites differentiate amnestic mild cognitive impairment from healthy controls and predict early alzheimer’s disease via untargeted lipidomics analysis. Front Neurol. 2021;12:704582. doi: 10.3389/fneur.2021.704582 PMC8365883 34408722

