# Supplementary material for: Oral microbiome–metabolome axis links glycerophospholipid dysregulation to Alzheimer’s disease
Source: J Oral Microbiol. 2026 Sep 29;18(1):2732297. doi: 10.1080/20002297.2026.2732297 (PMC13629805; doi:10.1080/20002297.2026.2732297)
Supplement: Supplementary file 1 [file ZJOM_A_2732297_SM0954.docx]

Supplementary Table 1 Phylum-level relative abundance analysis of TB microbiota

| Phylum-level | CON | AD |
| --- | --- | --- |
| *Firmicutes* | 38.49% | 30.85% |
| *Bacteroidetes* | 23.13% | 22.00% |
| *Proteobacteria* | 11.39% | 26.89% |
| *Actinobacteria* | 14.32% | 9.975% |
| *Fusobacteria* | 9.422% | 7.710% |

Supplementary Table 2 Genus-level relative abundance analysis of TB microbiota

| Genus-level | CON | AD |
| --- | --- | --- |
| *Prevotella* | 19.39% | 18.20% |
| *Veillonella* | 15.38% | 14.07% |
| *Neisseria* | 7.411% | 13.69% |
| *Streptococcus* | 10.01% | 7.759% |
| *Actinomyces* | 8.417% | 5.956% |
